# Do Menopausal Symptoms Affect the Relationship between Job Demands, Work Ability, and Exhaustion? Testing a Moderated Mediation Model in a Sample of Italian Administrative Employees

**DOI:** 10.3390/ijerph181910029

**Published:** 2021-09-24

**Authors:** Sara Viotti, Gloria Guidetti, Ilaria Sottimano, Lucia Travierso, Mara Martini, Daniela Converso

**Affiliations:** 1 Dipartimento di Psicologia, Università Degli Studi di Torino, 10124 Torino, Italy; ilaria.sottimano@unito.it (I.S.); lucia.travierso@unito.edu.it (L.T.); mara.martini@unito.it (M.M.); daniela.converso@unito.it (D.C.); 2 Dipartimento di Scienze Psicologiche, Della Salute e Del Territorio, Università di Chieti-Pescara, 66100 Chieti, Italy

**Keywords:** menopausal symptoms, work ability, job demands, exhaustion, aging workforce

## Abstract

(1) Background: The increasing presence of employed women undergoing menopause has stimulated a growing corpus of research highlighting the complex relationship between menopause and work. Nevertheless, little is known regarding the mechanism by which menopause affects work ability and work-related well-being. In order to fill this gap in the literature, the present study examines whether and how menopausal symptoms affect the relationship between job demands, work ability, and exhaustion. (2) Method: In total, 1069 menopausal women, employed as administrative officers in a public organization, filled out a self-report questionnaire. A moderated mediation analysis was carried out using the latent moderated structural (LMS) equation. (3) Findings: The findings of this analysis indicate that the indirect effect of work ability on the relationship between job demands and exhaustion is influenced by the exacerbating effect of menopausal symptoms on the relationship between job demands and work ability. Moreover, the conditional effect confirmed that women with high menopausal symptoms receive more exposure to the negative effects of job demands on work ability compared to women with low menopausal symptoms. (4) Conclusion: The present findings may help in addressing interventions to prevent negative outcomes for menopausal women and their organizations.

## 1. Introduction

Menopause is a physiological process that marks the end of the reproductive phase of a woman’s life [1]. This process entails a constellation of symptoms (e.g., hot flashes, sleep disturbances, and decreased physical strength) attributed to hormonal changes, which may vary considerably in terms of incidence and intensity across individuals [2]. On average, the menopausal transition begins between the ages of 48 and 55 years and typically lasts for four to eight years [3]. However, prior studies have revealed that women may also report menopausal symptoms after the end of this transition period [3].

Over the last decade, it has become increasingly common for women to undergo menopause while being employed. In Organization for Economic Cooperation and Development (OECD) countries, the employment rate of women is rapidly increasing, such that women are approaching the employment rate of men [4]. In many Western countries, older women represent an especially important portion of the total workforce. In 2010, the percentage of working women aged 55–64 years in Germany was 54%; in 2019, this percentage reached 70% [5]. In a similar vein, the percentage of employed women in the age range of 55–64 years in Italy has risen from 27% to 47% in only ten years (2010–2019) [6]. In 2019, approximately 60% of the US female population aged 55–64 years were employed [4].

The increasing presence of employed women undergoing menopause has stimulated a growing corpus of research. This research initially took a primarily medical perspective and has, more recently, incorporated a psychosocial perspective by highlighting the complex relationship between menopause and work [7].

On one hand, current research seems to indicate that working women tend to tackle menopausal transition more effectively than non-working women [8,9]. The biological and body changes involved in menopause have a deep impact at the psychological level; specifically, these changes may generate anxiety, depression, and stress and may also reduce self-efficacy and self-esteem [10,11,12]. In this context, work may help women to build psychological resources useful to adapt themselves to the new biological condition of menopause, thus favoring the process of identity redefinition that this transition may entail [13]. Jobs may allow for the development of such resources by providing opportunities for women to achieve intrinsic and extrinsic development by engaging in meaningful projects and exercising control [14,15].

On the other hand, empirical evidence has also shed light on hindrances in the workplace that may undermine women’s well-being during menopausal age. Regardless of whether they are actually in menopause, older women (e.g., over 50) are at a higher risk of being subjected to social stigma and targeted for workplace discrimination due to their (supposed) menopausal status [7,16]. Negative stereotypes, which are culturally rooted in many workplaces, characterize menopausal women as irrational, emotionally unstable, and non-performative and may work as self-fulfilling prophecies [17], thus impeding women’s abilities to fully express their potential at work. For example, the stereotype of the menopausal woman is incongruent with the expected characteristics of the leader; therefore, senior women who aspire to achieve leadership positions may be discarded, despite their experience in their fields of work [18]. This type of subtle discrimination represents an expression of the glass ceiling, which refers to gender inequalities in the workplace that represent an important obstacle to the development of women and their organizations [19].

Moreover, there is empirical evidence that diminished work ability can be a concrete risk for women undergoing menopause [15,20]. According to Ilmarinen, work ability refers to the physical and intellectual resources upon which workers rely to meet the demands posed by their jobs [21]. Studies have pointed out that approximately 25–30% of menopausal women report bothersome symptoms impairing their ability to work [22,23]. While work ability [24] tends to decrease significantly during menopause, work-related stress indicators (e.g., burnout [4,25]) tend to increase during menopause. More recent studies [26] found that unfavorable working conditions (e.g., low job autonomy, low social support, and no flexibility in working hours) are associated with decreased work ability among menopausal women.

Despite this rapidly growing body of literature on work ability, knowledge regarding the effects of menopausal symptoms on work ability is vague. In particular, the specific mechanism by which menopausal symptoms affect women’s abilities to respond to job demands and the outcomes of this process, especially in terms of work-related stress response, remains largely unknown. Indeed, there is a paucity of studies that have examined work-related stress response (e.g., burnout) among menopausal women [25], and no studies have examined in detail whether and how work-related stress among menopausal women may be explained by the possible modifications that may have occurred in the ability to respond to the demands posed by their jobs.

It is also important to highlight that there is evidence in contrast with the idea that menopause is necessarily associated with a decline in work ability [18]. According to Atkinson, the problems experienced by menopausal women may be unrelated to menopause and may arise for many reasons, both health-related and non-health-related (e.g., aging) [18]. Moreover, while menopausal symptoms are positively associated with work-related stress indicators and the inability to work, the most recent literature seems to indicate that menopausal stage or status is not associated with these factors [27,28]. A study by Geukes and colleagues [23] found that work ability might be impaired only in the case of severe menopausal symptoms. Overall, these studies indicated that the generalization of a non-specific menopause effect on work seems to be completely inappropriate and that to understand how menopause affects women’s work ability and well-being, it is important to focus on the specific menopausal symptomatology experienced by each woman rather than on menopausal status alone. Making menopause a major focus of this line of research without clarifying the real effects of menopausal symptoms on women’s abilities to respond to job demands could have the undesirable side effects of disempowering menopausal women and reinforcing negative stereotypes that characterize menopausal women as lacking and not fit for the lifestyle and demands of the workplace [16]. Based on this, the present study proposes that menopause interferes with work, depending on the cocktail of symptoms and their intensity, both of which vary considerably between individuals [29]. Specifically, the target hypothesis is that menopausal symptoms exacerbate the indirect relationship between job demands and exhaustion via work ability. From a definitory point of view, exhaustion is considered the *core* burnout symptom and represents the long-term end-state of the resource loss process in the workplace at the individual level [30].

The indirect effect of work ability in the relationship between job demands and exhaustion has been demonstrated by a previous study of aging female workers [31]. However, this subject has not been studied among menopausal working women or by accounting for the severity of menopausal symptomatology. The present study was conceived to fill this gap in the literature by shedding light on the role of menopausal symptomatology in modifying this indirect effect.

A contribution in this direction could have many practical implications. The resulting empirical evidence could help us dissipate the taboo surrounding menopause and contrast negative stereotypes with the true effects of menopause on work. Furthermore, this evidence could be used to promote the sustainability of work for women whose menopausal symptoms interfere with their work ability and work-related well-being, thus promoting interventions that can prevent negative outcomes not only for these women but also for their organizations.

## 2. Materials and Methods

### 2.1. Data Collection and Participants

Under an agreement between the Municipality of Turin and the Department of Psychology at the University of Turin, a survey of workers employed at the Municipality of Turin was carried out to assess work-related stress and well-being. In December 2017, a self-report questionnaire was sent to the institutional e-mails of all administrative employees at the Municipality of Turin. A question stating “Are you in menopause”? (response: “yes/no”) was used to identify menopausal women and invite them to fill out a further section of the questionnaire, which specifically examined job-related well-being and sustainability during menopause. In order to help these women respond correctly and to minimize the risk of false-positives, the aforementioned question was accompanied by the following statement: “While responding, please consider that menopause is diagnosed when a woman has gone without a menstrual period for 12 consecutive months”.

Of the 9531 questionnaires sent out, 3026 were returned and filled out correctly. Of the respondents who returned these questionnaires, 1069 declared that they were menopausal women. The final dataset of this study consisted of 1054 menopausal women who had correctly filled out the questionnaire. The average age of these participants was 56.24 years (SD = 6.32, min = 38 years, max = 66 years), and the average length of time in menopause was 6.32 years (SD = 4.55, min = 1 year, max = 26 years). All participants were administrative officers with permanent contracts and average job seniority of 28.91 years (SD = 8.01). With regard to health, 8.7% of the participants declared no diagnosed diseases, 10% declared one diagnosis, 12.3% declared two diagnoses, 19.6% declared three diagnoses, and 49% reported four or more diagnoses (20.1% = four diagnoses, 29.3% = six diagnoses).

The research protocol was developed in accordance with Italian Law 101/2018 on workplace privacy and conformed to the provisions of the 1964 Declaration of Helsinki (as revised in Fortaleza, 2013). All ethical guidelines for human research were followed in this study. The questionnaire was covered with a letter that openly described the research purpose and the anonymity of data collection and treatment. This cover letter also clearly stated the voluntary nature of participation in this study. All participants signed an informed consent form.

### 2.2. Measures

The questionnaire included the following scales and variables:

Job demands. Job demands were measured with the five-item quantitative demands scale of the Job Content Questionnaire (e.g., “I am asked to do an excessive amount of work”) [32,33]. Responses fell on a four-point scale, with a range of 1 (“strongly disagree”) to 4 (“strongly agree”).

Work ability. Work ability was assessed with one item from the Work Ability Index [34]. Respondents were asked to rate their current work ability compared to their best lifetime work ability. Responses fell on a scale ranging from 0 (“cannot currently work at all”) to 10 (“work ability at its best”).

Exhaustion. Exhaustion was measured with a five-item subscale of the Maslach Burnout Inventory-General Survey (MBI, [35]; e.g., “I feel burned out from my work”). All items were scored on a seven-point Likert scale, ranging from 0 (“never”) to 6 (“every day”).

Menopausal symptoms. Menopausal symptoms were measured with 24 items (e.g., “experiencing hot flashes”) from the Menopause-Specific Quality of Life (MENQOL; [36]) questionnaire. The original version of the MENQOL questionnaire has 29 items. However, under the assumption that a shorter questionnaire could encourage participation, three items measuring symptoms of the sexual sphere (e.g., “change in sexual desire”, “vaginal dryness during intercourse”, and “avoiding intimacy”) and two items measuring appearance-related symptoms (e.g., “change in appearance, texture, or tone of your skin” and “increased facial hair”) were not included. These items were not considered essential for the aims of this study, which are focused on studying how menopause affects the quality of life in the workplace. Moreover, respondents might have considered items in the sexual domain to be intrusive and overly intimate; therefore, the inclusion of these items could have discouraged respondents from completing the section on menopause. Both the research group and the organizational stakeholders involved in this research project (i.e., work representatives, occupational health specialists, and direction staff) agreed with this decision.

The literature on the psychometric structure of the MENQOL questionnaire is inconclusive. In the original version of the MENQOL questionnaire [36], 24 items were loaded on 3 subdimensions: the vasomotor (e.g., “hot flashes”), psychosocial (e.g., “experiencing poor memory”), and physical (e.g., “aches in the back of neck or head”) dimensions. However, later research yielded conflicting results. Nie et al. [37] proposed a four-factor structure in which physical symptoms would be loaded on two different dimensions: physical feeling (e.g., “decrease in stamina”) and physical ache (e.g., “frequent urination”) dimensions. Converso et al. [25] provided evidence for a one-factor structure in which all items would be loaded on one large, latent factor: menopausal symptoms. In light of these contrasting results, prior to testing the hypotheses, the present study employed confirmatory factor analyses (CFAs) to identify which structure would best fit the data. Each item was rated on a scale from 1 (“not present/not bothersome”) to 6 (“extremely bothersome”).

Control variables. The models were controlled by age, job seniority, length of menopause, and health problems, as the literature has recognized the influence of these factors on the major study variables [24,31,38]. To measure health problems, a checklist of 15 diseases from the WAI [34] was included in the questionnaire (range: 0 = “no disease” to 15 = “15 diseases”).

### 2.3. Data Analysis Strategy

All data analyses were carried out with SPSS (IBM, Armonk, NY, USA) and MPlus (Computer Software, Los Angeles, CA, USA). In order to ascertain the psychometric proprieties of the multi-item scales and the distinctiveness of their items (in light of the contrasting results regarding the number of dimensions underlying the MENQOL questionnaire), various combinations of different numbers of factors were compared. To accomplish this, a series of CFAs was performed using robust maximum likelihood (MLr) as an estimation method.

At Step 1, a one-factor model was specified, with all items loaded on a single factor (SBF); an acceptable fit of this model would likely indicate that common method bias is a major issue [39]. At Step 2, two factors were modeled: job demand items were loaded on one factor, while the remaining items were loaded on another factor (JD-SBF). At Step 3, a three-factor solution was modeled: while both job demand and exhaustion items were loaded on their corresponding factors, all menopausal symptom items were loaded on the same factor (JD-EX-Ms); this three-factor model, including the menopausal symptom factor, refers to a solution developed by Converso et al. [25]. In the four-factor model (Step 4), items regarding job demands, exhaustion, and menopausal psychosocial symptoms were specified to be loaded on their corresponding factors, while the remaining items were specified to be loaded on the same factor (menopausal physical symptoms) (JD-EX-MPSs-MPHs). In addition to job demands and exhaustion, the following three subdimensions of menopausal symptoms were specified in the five-factor model (Step 5): psychosocial, vasomotor, and physical symptoms (JD-EX-MVs-MPSs-MPHs); this partition of menopausal symptoms refers to the original version of the MENQOL questionnaire, as proposed by Lewis et al. [36]. Finally, in the six-factor model (Step 6), as indicated by Nie et al. [37], physical symptoms items were specified to load on two different factors: physical feeling and physical aches (JD-EX-MVs-MPSs-MPHFs-MPHAs).

**Figure 1:**
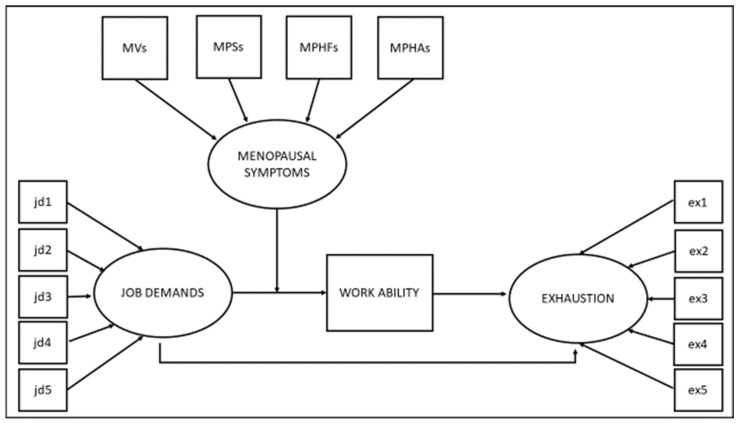
The model tested.

Pearson’s correlations were performed to check the significance and directions of the relationships between all the considered variables. A mediation analysis was carried out using a structural equation modeling (SEM) approach, and bias-corrected bootstrapping was utilized with 5000 bootstrap samples to estimate the confidence intervals of the indirect effects [40]. Finally, the LMS equation procedure developed by Sardeshmukh and Vandenberg [41] was used to simultaneously analyze the moderated and mediation hypotheses (see Figure 1 for a graphical representation of the hypotheses tested).

The fits of the CFA and SEM were assessed with the comparative fit index (CFI), the Tucker-Lewis index (TLI), the standardized root mean square residual (SRMR), and the root mean square error of approximation (RMSEA). For the TLI and CFI, values higher than 0.90 were considered indicators of a good model fit [42,43]. An SRMR value equal to or less than 0.09 also indicated a good model fit [44]. Finally, an RMSEA value lower than 0.08 indicated an acceptable model fit [45]. In addition, the Akaike information criterion (AIC) and Bayes information criterion (BIC) were used to compare the alternative (non-nested) measurement models [46]. The model with the lowest AIC and BIC was considered the best-fitting model.

## 3. Findings

### 3.1. Preliminary Analyses

The CFA analyses are reported in Table 1. The six-factor model, which considered all the multi-item major study variables (i.e., job demands, emotional exhaustion, menopausal vasomotor symptoms, menopausal physical feeling symptoms, menopausal physical ache symptoms, and menopausal psychosocial symptoms) as distinct factors, showed the best fit with the data (χ^2^ = 2488.17, df = 512, CFI = 0.90, TLI = 0.91, RMSEA = 0.05 [0.04–0.05], SRMR = 0.05). In this solution, all items were significantly loaded on their corresponding factors (0.60 > λ > 0.92). The six-factor model fit the data significantly better than any alternative model, including the one-factor model, in which all items were loaded on a single latent factor (χ^2^ = 9869.52, df = 527, CFI = 0.58, TLI = 0.55, RMSEA = 0.13 [0.12–0.13], SRMR = 0.10). These findings were also confirmed by the AIC and BIC, which reached their lowest values in the six-factor model out of all the tested models.

Table 2 reports Cronbach’s alphas and Pearson’s correlations between all the study variables (i.e., the major study variables and control variables). Internal consistency of all measures was found to be satisfactory since all multi-item scales reported alpha values ≥0.80. Job demands were significantly correlated with both work ability (r = −0.27) and exhaustion (r = 0.34). Furthermore, work ability and exhaustion were significantly correlated with each other (r = −0.31). All menopausal symptom subdimensions were strongly correlated with each other (0.74 > r > 0.89). However, no menopausal symptom factors were found to be significantly associated with any other major study variables.

Regarding the control variables, health problems were significantly associated with job demands (r = 0.17), work ability (r = −0.23), exhaustion (r = 0.33), and menopausal physical feeling symptoms (r = 0.07).

### 3.2. Hypothesis Testing

In the SEM and LMS, the four subdimensions identified through the CFA of menopausal symptoms were included as manifest variables loading on a single latent factor. This choice to build an aggregate-level indicator solution instead of a second-order factor solution for menopausal symptoms was made in consideration of the tight associations between the subdimensions highlighted by Pearson’s correlations. This choice also had the advantage of maintaining the favorable indicator-to-sample-size ratio of the overall model [47].

Before testing the study hypothesis, a mediated model was estimated using SEM analysis to examine the indirect effect of job demands on exhaustion via work ability. The partially mediated model obtained a satisfactory global fit: χ^2^ = 465.59, df = 42, CFI = 0.93; TLI = 0.92; RMSEA = 0.08 [0.08–0.10], SRMR = 0.03. Each item was significantly loaded on each corresponding factor (0.64–0.86). Job demands significantly affected work ability (β = −0.30, *p* = 0.0001), which, in turn, significantly affected emotional exhaustion (β = −0.23, *p* = 0.0001). Moreover, the relationship between job demands and emotional exhaustion was found to be significant (β = 0.32, *p* = 0.0001), suggesting that work ability partially mediated this relationship. This finding has been further confirmed by the estimation of direct (b = 0.90, *p* = 0.0001; CI = 0.63–1.631) and indirect (b = 0.20, *p* = 0.0001; CI = 0.10–0.30) effects, which were both statistically significant. After adjusting for control variables (i.e., age, health problems, years of menopausal status, and job seniority), the partially mediated model was confirmed. This model obtained a satisfactory global fit (χ^2^ = 533.69, df = 74, CFI = 0.93; TLI = 0.91; RMSEA = 0.08 [0.07–0.08], SRMR = 0.03) and confirmed the significance of both direct (b = 0.78, *p* = 0.0001; 99.5% CI = 0.56–1.06) and indirect (b = 0.14, *p* = 0.0001; 99.5% CI = 0.06–0.25) effects. With regard to the single paths, job demands significantly affected work ability (β = −0.27, *p* = 0.0001) and emotional exhaustion (β = 0.28, *p* = 0.0001); moreover, work ability significantly affected emotional exhaustion (β = −0.18, *p* = 0.0001), suggesting that work ability partially mediated the relationship between job demands and exhaustion. Among the control variables, health problems only had significant effects on the major study variables, showing a positive relationship with job demands (β = 0.18, *p* = 0.0001) and exhaustion (β = 0.26, *p* = 0.0001) as well as a negative relationship with work ability (β = −0.18, *p* = 0.0001).

LMS equation procedure was employed to test the mediated moderation hypothesis (H2). In this model, each latent variable was significantly loaded on its corresponding factor (*p* = 0.0001). The relationships between the main terms—job demands (b = −0.32, *p* = 0.0001) and menopausal symptoms (b = 0.24, *p* = 0.0001)—and work ability were significant. The interaction term between menopausal symptoms and job demands showed a significant influence on work ability (b = −0.12, *p* = 0.004). Moreover, work ability was significantly associated with exhaustion (b = −0.25, *p* = 0.0001), and job demands significantly affected exhaustion (b = 0.98, *p* = 0.0001). The conditional effect, which was calculated using a bootstrap procedure, showed that the indirect effect of job demands on exhaustion via work ability was not significant when menopausal symptoms were low (b = −0.05; *p* = 0.33; 99.5% CI = −0.19–0.08). Conversely, this indirect effect was significant when menopausal symptoms were high (b = 0.11; *p* = 0.02; 99.5% CI = 0.04–0.24).

After adjusting for control variables (i.e., age, health problems, length of menopause, and job seniority), the above reported findings were fully confirmed. The effects of job demands (b = −0.36, *p* = 0.003) and menopausal symptoms (b = 0.11, *p* = 0.0001) on work ability were significant. The interaction term between menopausal symptoms and job demands was significant as well (b = −0.10, *p* = 0.02). Finally, work ability was significantly associated with exhaustion (b = −0.20, *p* = 0.0001), and job demands significantly affected exhaustion (b = 0.79, *p* = 0.0001).

Regarding the control variables, health problems significantly affected menopausal symptoms (b = 0.78; *p* = 0.0001), workload (b = 0.05; *p* = 0.0001), work ability (b = −0.06; *p* = 0.02), and exhaustion (b = 0.21; *p* = 0.0001). The length of menopausal status was significantly negatively associated with menopausal symptoms (b = 0.04; *p* = 0.04).

**Figure 2:**
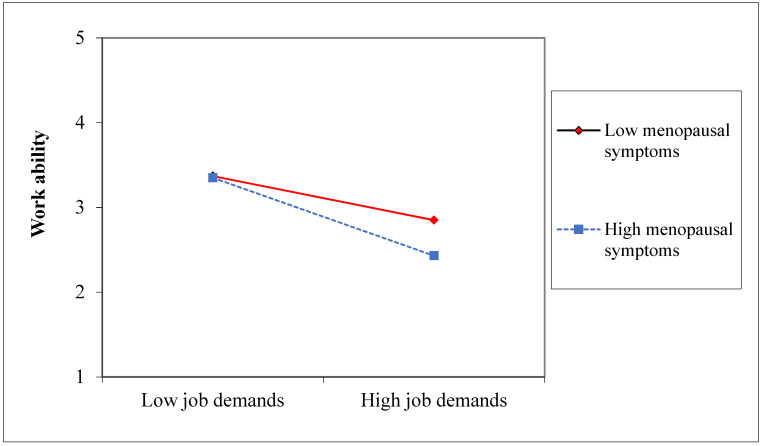
The moderating effect of menopausal symptoms in the relationship between job demands and work ability.

The conditional effect (Figure 2) indicated that the indirect effect of job demands on exhaustion via work ability was not significant when menopausal symptoms were low (b = 0.01; *p* = 0.89; 99.5% CI = −0.10–0.09). Conversely, this indirect effect was significant when menopausal symptoms were high (b = 0.10; *p* = 0.02; 99.5% CI = 0.03–0.23).

## 4. Discussion

### 4.1. Theoretical Implications

The present study examines the effect of menopausal symptoms on the mediating role of work ability in the relationship between job demands and exhaustion in a sample of working women undergoing menopause. In particular, it was hypothesized that the indirect effect of work ability in the relationship between job demands and exhaustion is influenced by the moderating effect of menopausal symptoms on the relationship between job demands and work ability.

The present analyses support our hypotheses. Specifically, the findings showed that the indirect effect of work ability on the relationship between job demands and exhaustion was significant only in cases of high menopausal symptoms. Conversely, this indirect effect was not significant when menopausal symptoms were low. This finding suggests that menopausal symptoms, rather than menopausal status alone, exacerbate the loss spiral initiated by job demands. In this regard, this study demonstrates that menopause alone does not necessarily represent a hampering condition or negatively affect women’s ability to cope with job demands. Furthermore, this finding is compatible with those of previous works, which have sustained that menopausal women may perform better than their younger colleagues [7,17].

On the other hand, this study highlights a serious risk to menopausal women reporting menopausal symptoms. In the interaction with job demands, menopausal symptoms may overtax the psychophysiological system and dysregulate the individual’s energy balance by hampering work ability [31]. As highlighted by previous studies, work ability tends to decrease with age; it is unlikely that this lost work ability can be physiologically recovered, especially for those aged over 50 years [48]. Although the symptoms of menopause tend to represent a temporary transition, their consequences on the work domain may not be transitory and may, thus, have long-term effects on women’s work-related physical and psychological well-being.

Overall, our findings confirm previous studies that have suggested that the generalization of a non-specific menopause effect on work seems to be completely inappropriate [17,18,19]. Moreover, it also suggests that in order to understand how menopause affects women’s work ability and well-being, it is important to employ specific measures capable of detecting the intensity of specific menopausal symptomatology experienced by women.

### 4.2. Practical Implications

Overall, the present findings have several practical implications. Firstly, these findings suggest that organizational management should develop an awareness that the menopause experience and its effects on work vary considerably across different women. Therefore, it is important to avoid basing new policies and interventions on the implicit a priori generalization that menopause is a problematic condition. One potential risk of taking this position, not far from gendered ageism and stigmatization, is the medicalization of all women undergoing menopause. Conversely, a data-driven, bottom-up approach could be an effective means of identifying menopausal women’s specific needs in the workplace. Surveys using self-report questionnaires, individual interviews, or focus groups can be employed to involve workers in participatory processes, with the aim of identifying interventions that may make workplaces more menopause-friendly. Additionally, occupational physicians should be involved in the development of a monitoring system to identify individual cases of women with severe menopausal symptoms who require specific interventions (e.g., temporary flexible working hours or workload reduction). Such interventions could preserve work ability, thus containing the risk of a loss spiral, which would be detrimental for menopausal women.

The present findings also highlight that in order to effectively manage menopause in the workplace, it is important to develop a prevention strategy at both organizational and public-institutional levels. In particular, the development of training, policies, and activities specifically related to menopause may be crucial to improve women’s job sustainability across their entire working lifespans. Examples include information campaigns with the aim of generating a cultural environment that is more positive toward the specific topic of menopause at work. Health promotion programs should include information about menopause, aging, and health to help women adopt healthier lifestyles (e.g., diet changes, stress management, and the development of positive attitudes toward aging and menopause). There is a large corpus of studies that has provided evidence for the effectiveness of health lifestyle training in promoting health and preventing specific risks for menopausal women. In particular, introducing healthy behaviors such as practicing regular aerobic and resistance exercise and modifying diet, avoiding alcohol and nicotine, might improve quality of life and cognitive function and reduce cardiovascular disease, osteoporosis, and metabolic syndrome risks [49,50,51,52,53,54]. Moreover, actions specifically directed at increasing personal resources (e.g., self-efficacy, optimism, and resilience), such as offering mindfulness classes or psychological support services, may help menopausal women better tackle the transition process of menopause. Evidence from randomized controlled trials has demonstrated the effectiveness of the Mindfulness-Based Stress Reduction (MBSR) protocol in reducing menopausal symptoms and psychological diseases such as anxiety and depression [55,56]. Finally, any specific organizational policies or interventions in the work environment (e.g., job design or ergonomic adjustments), with the aim of improving employees’ quality of work-life, may promote job sustainability by preserving the health status of all workers, including menopausal women.

### 4.3. Direction for Future Research

Using these encouraging findings, we have identified several recommendations for future research that may further the development of knowledge in this field. As mentioned previously, the present study examined the manner in which menopausal symptoms interfere with work. However, seminal work has suggested that the work environment may affect menopausal symptoms [23]. Therefore, future studies may use a cross-lagged design to test the reciprocal relationship between work and menopause.

Another issue that needs more attention from researchers in relation to menopause is gendered ageism and discrimination [7,24]. Whereas studies suggest that menopausal women are exposed to this type of risk, little is known about their consequences on job well-being. Therefore, future studies should examine whether and how exposure to ageism may affect the job-related well-being, work ability, and performance of women undergoing menopause.

### 4.4. Limitations

The most relevant limitation of the present study is its cross-sectional design. Future research should employ a longitudinal design to explore the cross-lagged associations between the examined constructs. Longitudinal studies may also be useful for understanding whether and how the relationships between these constructs change over time.

Another limitation of this study is that it only employed self-report measures. The use of only a single data source may introduce the issue of common method variance. Future studies may benefit from research designs including a combination of objective measures (e.g., medical diagnosis of menopausal syndrome) and subjective measures or data from multiple sources (e.g., a job analysis to assess job demands).

Moreover, the representativeness of the results may have been another limiting factor. The present study only surveyed one specific professional group (i.e., administrative officers). Therefore, caution should be exercised when generalizing the results to menopausal women employed in other occupational sectors.

Finally, a possible limitation may be the employment of a single-item scale to measure work ability. The single-item measure for this construct is usually employed in research mostly because it contributes to a reduction in time of completion and makes the questionnaire easier to fill out. However, it is important to highlight that besides evidence supporting the reliability of a work ability single-item measure [57,58], there are also studies that prefer multi-item measures, which are considered more robust [59].

## 5. Conclusions

Although the urgency of identifying work-related risks for women undergoing menopause has largely been recognized, this is the first study (to the best of our knowledge) that has tried to understand the specific mechanism by which menopausal symptoms affect the relationship between job demands, work ability, and exhaustion. In this respect, the present findings bring to light the complex relationship between the characteristics of the work environment, job-related well-being, and menopause. As hypothesized, it was found that menopausal symptoms represent a health-impaired condition that activates the potential of job demands to serve as a hindrance, leading to the loss of work ability and, thus, exhaustion.

## Figures and Tables

**Table 1:** Confirmatory factor analyses (CFA): test of alternative models (goodness-of-fit indices).

	χ^2^	Df	CFI	TLI	SRMR	RMSEA [CI]	AIC	BIC
M6. Six-factor model (JD-EX-MVs-MPSs-MPHFs-MPHAs)	2488.17	512	0.90	0.91	0.05	0.06 [0.06−0.06]	113,203.46	113,800.29
M5. Five-factor model (JD-EX-MVs-MPSs-MPHs)	3075.44	517	0.88	0.87	0.07	0.07 [0.07–0.08]	113,905.93	114,477.88
M4. Four-factor model (JD-EX-MPSs-MPHs)	4675.62	521	0.81	0.79	0.07	0.08 [0.08–0.09]	115,848.48	116,400.55
M3. Three-factor model (JD-EX-Ms)	6613.32	524	0.73	0.71	0.08	0.10 [0.10–0.10]	118,233.64	118,770.78
M2. Two-factor model (JD-SBF)	8502.74	526	0.65	0.63	0.09	0.11 [0.11–0.12]	120,185.10	120,712.29
M1. One-factor model (SBF)	9869.52	527	0.58	0.55	0.10	0.13 [0.12–0.13]	122,294.65	122,816.88

Note: JD = job demands. EX = exhaustion. MVs = vasomotor symptoms. MPSs = menopausal psychosocial symptoms. MPHFs = menopausal physical feeling symptoms. MPHAs = menopausal physical aches symptoms. Ms = menopausal symptoms. SBF = single big factor. Df = degree of freedom. CFI = comparative fit index. TLI = Tucker-Lewis index. SRMR = standardized root mean square residual. RMSEA = root mean square error of approximation. AIC = Akaike information criterion. BIC = Bayes information criterion.

**Table 2:** Descriptive analyses and Pearson’s correlations.

	M(sd)	A	1	2	3	4	5	6	7	8	9	10
1. Job demands	7.43(2.43)	0.86	1									
2. Work ability	7.65(1.40)		−0.27 **	1								
3. Menopausal vasomotor symptoms (MVs)	5.62(5.38)	0.92	−0.01	−0.011	1							
4. Menopausal psychosocial symptoms (MPSs)	8.73(6.44)	0.94	0.01	−0.03	0.86 **	1						
5. Menopausal symptoms (MPHFs)	18.97(11.14)	0.92	0.01	−0.06	0.86 **	0.74 **	1					
6. Menopausal physical symptoms (MPHAs)	11.17(6.72)	0.80	0.00	−0.04	0.89 **	0.86 **	0.86 **	1				
7. Exhaustion	12.57(8.57)	0.92	0.34 **	−0.31 **	−0.01	0.00	0.04	0.03	1			
8. Age (years)	56.24(3.82)		−0.03	−0.03	−0.00	−0.00	−0.01	0.01	0.04	1		
9. Job seniority (years)	28.91(8.00)		−0.01	0.02	−0.01	0.00	0.00	−0.01	0.05	0.49 **	1	
10. Length of menopause (years)	6.34(4.53)		−0.02	−0.04	−0.03	−0.02	−0.03	−0.01	0.02	0.53 **	0.26 **	1
11. Health problem (general)	1.86(1.80)		0.17 **	−0.23 **	0.02	0.01	0.07 *	0.04	0.33 **	0.06 *	−0.00	0.03

Notes. ** ≤0.001; * ≤0.05.

## Data Availability

The data that support the findings of this study are available from the corresponding author, upon reasonable request.
